# Supplementary material for: Are carbon emissions trading and green financial instruments synergistic? -Comprehensive quantitative research based on content analysis
Source: PLoS One. 2024 Mar 7;19(3):e0298601. doi: 10.1371/journal.pone.0298601 (PMC10919601; doi:10.1371/journal.pone.0298601)
Supplement: S1 Data — (ZIP) [file pone.0298601.s001.zip › Data/地级市Z_F工作报告.zip!/╔╜╬≈/╘╦│╟╩╨/└┤╫╘╣1⁄2╓┌║┼í╛┬φ┐╦╩2╛▌═°í┐_2015.docx]

各位代表：

　　现在，我代表市人民政府向大会报告工作，请予审议，并请市政协委员和其他列席人员提出意见。

　　一、2014年工作回顾

　　2014年是运城发展史上非同寻常的一年。面对特殊历史时期和经济下行压力加大的严峻形势，全市人民紧紧围绕中央、省、市的各项决策部署，团结奋进，共克时艰，保持了全市经济持续健康发展和社会和谐稳定。

　　全市主要经济指标增幅在全省排位大幅前移：全年地区生产总值完成1201.6亿元，增长5%；规模以上工业增加值完成386.9亿元，增长3.8%；固定资产投资完成1202.7亿元，增长19.2%；社会消费品零售总额完成620.3亿元，增长12.3%；财政总收入完成101.7亿元，增长11.4%，历史性突破百亿大关；公共财政预算收入完成52.8亿元，增长16.2%；城镇居民人均可支配收入完成22226元，增长8.9%；农村居民人均可支配收入完成8125元，增长11.5%。全市固定资产投资、公共财政预算收入、城镇居民人均可支配收入等3项指标增速排名均居全省第一；农村居民人均可支配收入、社会消费品零售总额增速分别排名全省第二、第三。

　　这一年，我们提升发展理念，强化顶层设计，推动运城转型发展的路径更加科学明晰。我们制订完善了工业集群化、农业现代化、新型城镇化“三个方案”，市人大常委会作出决议，使“三个方案”转化为全市人民的共同意志，成为今后一个时期内引领运城经济社会发展的纲领性文件，成为统筹工业与农业、城市与乡村、经济与社会发展的基本路径。工业集群化全面入轨、成效明显。“全市经济一盘棋，错位发展、差异竞争、优势互动”的发展理念深入人心，九大产业集群完成工业总产值915.7亿元，占到全市工业的57%，同比增长7%，增速高于全市工业平均水平9.6个百分点。26个主攻产业板块实施项目239个，落地资金972亿元，完成投资281亿元。产业集群成为全市工业经济发展的主引擎。创办小微企业2050户，培育“小升规”企业46户，培育“小巨人”企业18户。具有运城特色的农业现代化之路正在拓宽。以土地规模化打造优势基地特色板块，以组织企业化培育新型农业经营主体，以技术现代化建设高效型科技农业，以经营市场化建立农业生产、流通和服务体系，加快推进农业大市向农业强市转变，农业现代化建设取得了新进展。全年新增土地流转面积81.9万亩，累计达到204.6万亩。现代农业示范园区发展到259个。各类专业合作社累计达到1万家，总数位居全省第一。农产品加工企业实现销售收入230亿元。夏县润恒、芮城大汉等农产品物流项目加快建设。农产品电子商务蓬勃发展，全年通过电子商务平台实现农产品销售收入上亿元。全市粮食总产量31.6亿公斤，创历史最高水平。传统农业地区新型城镇化步伐加快。我们以工业集群化、集群园区化、园区社区化、社区城镇化、土地规模化、城乡生态化“六化”衔接为路径，推动工业化、信息化、城镇化、农业现代化“四化”互动发展，坚持中心城市、大县城、小城镇、新农村“四位一体、功能各异”，优化新型城镇化空间布局。以“城市功能完善、产业空间拓展、土地集约利用、市民方便宜居”四个目标一个过程实现为原则，着力提升中心城市辐射带动作用。在建项目106项，完成投资42.4亿元。10个旅游酒店、10个产业大厦、10个商业综合体开工建设15个。市民期盼多年的河东西街延长线工程得以启动，力争今年“十一”具备通车条件。大县城着眼于通过产业集聚，实现人口集聚的功能，狠抓公共服务设施建设，实施在建项目203个、完成投资86.5亿元。小城镇着眼于繁荣农村市场和提升农村公共服务功能，15个重点镇建设顺利推进，完成投资5.4亿元。全市有20个建制镇入围全国重点镇名单。新农村着眼于推动土地规模经营和作为“一村一品”空间节点的功能，启动了26个美丽宜居示范村建设。

　　这一年，我们坚持精准发力，狠抓工作落实，推动各项重点工作的专业化水平有了明显提升。围绕“三个方案”的实施，我们还出台了信息化、高技能人才培训基地建设、国家创新驱动发展战略运城行动计划等多个方案，政府各部门对专业化的认识和工作专业化水平明显提高，创造性推动工作的能力不断增强。集群化、专业化、小分队、产业链招商成绩斐然。全年签约项目总金额达到3422亿元，完成率全省第一；到位资金691亿元，完成率全省第一，保证了我市投资增长全省第一，并且投资总额历史性地跃居全省第四。信息化建设稳步推进。编制了《运城市信息化发展五年规划》，与深圳中兴集团合作的智慧城市建设全面启动。与移动、联通、电信合作的“宽带运城”项目建设进展顺利。我市被国家列为首批信息惠民试点城市。万荣县智慧城乡系统平台成功运行。城乡生态化建设成效明显。按照“山上治本、身边增绿、林业增效、产业富民”的思路，持续加大植树造林力度，完成造林41万亩，新增绿化面积290万平方米。全年完成节能技术改造项目50个，空气质量大幅改善，省下达的节能降耗和污染防治任务圆满完成。涑水河重点段河道治理工程全面开工。“古中国”使运城文化旅游产业发展的主题更加鲜明，定位更加准确。按照“高端创意、整合资源、政府引导、市场运作”的思路，提出了以“打造古中国为标识的国际旅游目的地”的战略构想。组织召开了由北京大学等国家级权威机构专家参加的研讨会，“古中国”概念得到学术界的高度认同和旅游界的强烈反响，激发了旅游开发商的投资热情。关圣文化建筑群申遗完成了申遗文本编制和申报审核，建筑群周边环境整治工作全面开展。鼓楼修复主体工程已经完工，盐池东、中、西三禁门及部分禁墙修复保护工程全面展开。旅游产业快速增长，全年实现旅游总收入268.8亿元，增长26.4%。重点项目建设扎实推进。实施在建项目1197个，266项省市重点项目完成投资859.5亿元。大运汽车1万辆专用车、亚宝药业新生产线、大西高铁运城段等一批重点项目陆续建成。在全省“六位一体”考核指标排名中，我市有5项指标位居全省前3名，综合考核排名全省第一。

　　这一年，我们全面深化改革，努力扩大开放，加快发展的动力和活力进一步增强。各项改革稳步推进。深入推进行政体制改革和行政审批制度改革，承接落实省政府取消和下放的行政审批事项105项，市政府自主取消和下放行政审批事项71项。推行预先审办，实行联审联批，加强电子监察，行政审批时效提速30%。工商登记制度改革全面实施，新登记注册市场主体2.4万户，增长15.1%。食品药品监管体制改革深入推进，乡镇一级食药监管机构组建运行。黄河金三角区域合作持续深化。出台了《运城市贯彻落实晋陕豫黄河金三角区域合作规划实施方案》。成立了晋陕豫黄河金三角宣传思想文化工作联盟、果业产业发展协会等合作组织，先后签订了检验检疫合作备忘录、全面加强旅游合作协议、智慧物流城项目框架协议等12项合作机制。综改试验区建设全面加快。对外开放水平不断提升。对外贸易稳步增长，全市完成进出口总额14.98亿美元，出口完成4.8亿元，总量位居全省第二，对外贸易国家或地区达到121个。对接上海自贸区，与中金国泰集团合作，投资116亿元的黄河金三角智慧物流城项目正式落地。运城海关、出入境检验检疫局即将投入运营。

　　这一年，我们大力发展社会事业，着力保障和改善民生，人民福祉得到新改善。全年财政总支出的84.7%用于民生。在财力极其紧张的情况下，我们本着对事业和人民群众负责的态度，下决心解决政府历年的民生欠账。在2013年偿还1.7亿元的基础上，去年又偿还了2.8亿元，特别是把教师和医生的工作待遇作为涉及千家万户的基本民生问题来解决，优先理顺了长期遗留的700余名专业人员的基本待遇，保障了教师和医护人员队伍稳定。足额落实了省政府下达的调资政策。全年新增15家高新技术企业，新增有效发明专利70件。新改扩建公办标准化幼儿园28所，魏风小学主体完工，大运幼儿园、小学、初中，盐湖区实验小学北校、幼儿园完成规划；中等职业教育免学费和助学金政策全面落实，惠及学生10.5万人次；市实验中学参加全国第二届“中国汉字听写大会”荣获一等奖，向全国人民充分展示了运城崇教尚学的良好传统和基础教育的扎实成果。全市新农合参合率99.35%，人均政府补助标准达320元；新农合大病保险全面推行；12家县级医院完成远程会诊系统建设；投资10.5亿元的运城市第一医院开工建设，投资9000万元的市儿童医院正式启动。市群艺馆等公共文化基础设施建设档次提升，“戏曲惠民”、电影免费下乡受益群众300余万人次；新增国家级非物质文化遗产6项，名列全国地市级前茅。文化工作和“扫黄打非”工作均受到国家表彰。档案工作基础扎实，新建7个县级综合档案馆。全民健身活动蓬勃发展，竞技体育成绩显著。实施精准扶贫，百企千村产业扶贫工程投资23亿元，3.3万人稳定脱贫。城乡就业水平进一步提高。全市城镇新增就业5.8万人，城镇登记失业率2.57%。高技能人才培训基地建设稳步推进，开展各类培训13万人次。社会保障工作不断加强。全面落实社会保障与物价上涨联动机制，城乡低保、农村五保、企业养老保险、城乡居民养老保险和医疗保险各项领取标准稳步提高。新建老年人日间照料中心200家。农村“五件实事”全面完成。安全生产形势持续稳定好转。全市各类安全生产事故起数和死亡人数未超出控制指标。食品安全放心城市创建工作稳步推进。

　　这一年，我们推进依法治市，改进社会治理方式，民主法治和精神文明建设进一步加强。我们坚持“想透说清干实，尊重规律抓落实；有识有谋有勇，敬畏法纪敢担当”的行政理念，提升思维方式，改进工作方法。自觉接受人大法律监督、政协民主监督和社会舆论监督，认真听取各民主党派、工商联和无党派人士意见，支持工会、共青团、妇联等人民团体发挥作用。深入开展“六五”普法。圆满完成了第十届村委会换届选举。精神文明建设不断加强。荣获“山西省文明城市”称号。加强学习型政府建设，定期举办“政府大讲堂”活动，引导广大干部特别是领导干部不断转变观念、开阔视野、提升能力。严格执行中央“八项规定”、坚决反对“四风”，着力解决人民群众反映强烈的突出问题。大力压减“三公”经费，加大行政监察和审计监督力度，推进党风廉政建设和反腐败斗争，严肃查处违纪违法案件。社会治安防控治理全面加强，视频监控基本实现全覆盖。公安机关工作走在全省前列。信访维稳工作在特殊时期取得全省第一。认真贯彻党的民族宗教政策，民族团结、宗教和谐局面进一步巩固。国防后备力量建设持续加强，双拥工作和军民融合式发展深入推进。防震减灾、外事侨务、统计、文物、人防、地方志等工作不断加强，妇女儿童、老龄、残疾人等各项社会事业也取得新成绩。

　　各位代表，2014年，我们面对特殊困难，保持定力、负重奋进、成绩显著。这得益于我们自觉地、忠诚地、准确地、创造性地贯彻落实党中央、国务院和省委、省政府一系列决策部署；得益于市四大班子和全市人民同心同德，精诚团结，砥砺前行；得益于“三个方案”的顶层设计，广大干部明确了干什么、为什么、怎么干；得益于我们把理顺人心、稳定人心、凝聚人心作为促进改革发展稳定和加强干部队伍建设的核心工作，全市上下始终保持了干事创业的精神状态。在此，我代表市人民政府，向全市广大干部群众，向大力支持政府工作的人大代表、政协委员、各民主党派、工商联、无党派人士和人民团体，向驻运部队、武警官兵和公安民警，向所有关心支持运城发展的社会各界人士，表示衷心的感谢和崇高的敬意！

　　各位代表，我们也清醒地认识到，我市经济社会发展中还存在不少困难和问题，主要是：面对经济发展新常态，我们对运城面临的挑战和机遇认识还不够深刻，推进发展的视野和思路还不够开阔，应对经济结构深刻调整的办法还不多；社会事业欠账较多，民生改善任务繁重，维护社会稳定的压力较大；政府运转机制需要进一步优化，主动服务意识、依法办事意识和创造性开展工作的能力需要进一步提高，一些公务人员还存在较为严重的“为官不为”的消极表现；反腐败斗争形势依然严峻复杂，不敢腐、不能腐、不想腐的长效机制远未形成等。对这些问题我们一定在今后的工作中一步一个脚印，切实加以解决。

　　二、2015年工作安排

　　2015年是全面完成“十二五”规划目标任务的收官之年，也是我市实施“三个方案”、全面推动“六大发展”的起步之年。

　　今年政府工作的总体要求是：深入贯彻落实党的十八大、十八届三中、四中全会和全国、全省两会精神，按照中央“四个全面”战略布局和省委“六大发展”的总体要求以及市委三届六次全会的精神，坚持稳中求进工作总基调，积极适应经济发展新常态，以落实“三个方案”为主导，以扩大投资为抓手，以提高经济增长的质量和效益为目标，全面深化改革，努力扩大开放，突出创新驱动，加强法治建设，统筹推进工业集群化、农业现代化、新型城镇化、信息化、文化旅游产业和民生改善等重点工作，促进经济平稳健康发展和社会和谐稳定。

　　按照这样的思路，2015年上半年在宏观经济形势压力继续加大的情况下，全市各项工作全面铺开、有序推进，主要经济指标在全省继续保持较好水平。“三个方案”深入人心，推进有力。工业集群化优势日趋显现，园区功能进一步完善，九大产业集群链条不断延伸，新兴产业投资总量位居全省第一；农业现代化步伐坚实，投资大幅增长，小麦喜获丰收，成为历史上又一个高产年，苹果代表中国首次打入美国市场；具有运城特色的传统农业地区新型城镇化道路稳健起步，产城融合、产镇融合、产村融合的态势已经显现；改革开放深入推进，永东股份中小板上市，长荣农科、寰烁科技新三板挂牌，100家企业陆续推进股份制改造；“古中国”文化旅游品牌市场效应凸显；黄河金三角区域合作步伐加快。下一步，我们将按照既定的目标，坚定信心，狠抓落实，确保全年各项任务圆满完成。

　　综合考虑各方面因素，2015年我市经济社会发展的主要预期指标是：地区生产总值增长6.5%左右，全社会固定资产投资增长17%以上，社会消费品零售总额增长10%，公共财政预算收入增长5%，城镇居民人均可支配收入增长8%，农村居民人均可支配收入增长8%，城镇新增就业岗位5.6万个，城镇登记失业率控制在4.2%以内，居民消费价格涨幅控制在3%左右。

　　约束性指标是：全市万元地区生产总值能耗下降2.5%，万元地区生产总值二氧化碳排放量下降3%，化学需氧量、氨氮分别下降3.92%、3.99%；二氧化硫、氮氧化物分别下降2.37%、4.81%；烟尘和工业粉尘分别下降0.25%和0.5%。万元工业增加值用水量下降5%。

　　从上半年宏观经济情况看，要完成以上目标，下半年，我们需要付出极其艰辛的努力。

　　2015年，重点做好八个方面工作：

　 （一）以园区化发展、集群化招商为路径，加快工业结构优化升级

　　做大做强九大产业集群30个左右产业板块。继续落实每一个产业板块“当年新上项目不低于10个，落地资金不低于30亿元，当年完成投资不低于10亿元”的目标任务。大力推行市县两级领导、市直部门包联重点企业、包联九大产业集群和挂牌督办责任制。突出产业集群在工业经济发展中的考核分值和权重，传导压力，激发动力。

　　加大产业集群龙头企业扶持和引进力度。每个产业集群重点扶持1个—2个龙头企业，带动产业集群实现横向配套、纵向延伸。今年安排2亿元扶持基金，主要用于对产业集群的信贷融资奖励、信贷融资风险补偿、中小微企业发展等。重点抓好大运重卡、亚宝药业、阳光焦化、复晟铝业、华泽铝电、永济新时速、阳煤丰喜、恒晟纺织等龙头企业的同时，注重引进新的龙头企业入驻园区，引领集群。

　　深化产学研合作。加强与中科院北京分院、西北工业技术研究院、西北工业大学等科研院所的合作，真正把这些科研机构作为产业集群延伸产业链条、增强竞争实力、提高市场话语的外脑，提升集群创新能力。大运、阳光、亚宝、银光等企业要在新能源汽车研发、焦化行业环保技术升级、生物医药开发、高精密复杂铸件研发等方面发挥技术引领作用。市级企业技术中心增加5户，省级企业技术中心增加2户，青山化工要积极申报国家级企业技术中心。围绕九大产业集群，培育工业化、信息化“两化”融合示范标杆，大运、中铝山西分公司要争做全国“两化”示范企业。

　　突出抓好传统产业改造提升。围绕做大做强河津、永济、平陆、盐湖区4个铝工业园区，加快煤电铝材一体化进程，努力打造国家级铝工业示范基地。要突出抓好焦化、钢铁等重点行业改造提升，努力实现海鑫钢铁破产重整，提高传统产业竞争力。

　 （二）以土地规模化、组织企业化、技术现代化、经营市场化为路径，加快推进农业现代化

　　按照中央农村工作会议精神要求，认真落实我市2015年农业现代化推进意见，推进县一级农业产业板块发展项目计划，着力开展好农业产业招商引资，抓好459个农业现代化建设项目，突出典型带动，激发农业发展活力。

　　土地规模化方面，以土地确权登记为抓手，按照“依法、有偿、自愿”的原则，有序开展土地流转，着力打造优势基地和产业板块。稳步推进土地确权登记颁证工作，积极引导多种形式的农村土地适度规模经营。重点抓好粮食标准化生产、优质水果标准化生产、畜牧水产标准化养殖、无公害蔬菜标准化、农产品加工“五大基地”和干果经济林、中药材产业、食用菌产业、优质苗木、休闲观光农业“五大板块”以及现代农业示范区建设。完成粮食高产万亩创建示范片65个，建设水果示范园223个，新建改扩建标准化养殖场80个，新建设施蔬菜集约化育苗场5个，发展30家规模中药材专业合作社和1个—3个年产100万公斤以上的食用菌生产基地，培育1个—3个省级休闲农业与乡村旅游示范点。做好农业综合开发工作，建设高标准农田10.6万亩，完成生态治理6500亩。坚持精准扶贫，实施产业扶贫、科教扶贫和移民搬迁等重点项目，实现20个贫困村5.42万贫困人口稳定脱贫。

　　组织企业化方面，以培育新型经营主体为抓手，着力打造农产品产业集群。加快构建以农户家庭经营为基础、合作与联合为纽带、社会化服务为支撑的现代农业经营体系。大力扶持专业大户、家庭农场、农民专业合作社等，培育示范合作社200家，新认定家庭农场300家，新培育典型示范农场50家。通过点对点、小分队等多种形式，加大农业项目的招商引资力度。大力扶持农产品加工龙头企业，延伸产业链，提高附加值，扶持100家龙头企业，培育100家骨干企业，发展200家潜力企业，形成梯次合理的农产品加工业发展格局。全年农产品加工企业销售收入增长8%。大力发展现代畜牧业，着力推进养殖业提档升级、提质增效。积极引导永济粟海、闻喜象丰、夏县晋星为主的肉鸡企业拉长链条，做大做强；扶持稷山晋龙为主的蛋鸡企业，扩张规模，增强带动能力；大力发展生猪产业，以芮城温氏、永济长荣为龙头，加快基地建设，示范带动周边养殖户，搞好标准化养殖。积极与正大、大北农、山西大象等企业集团对接，争取年内有2个—3个大型畜牧项目签约落地。

　　技术现代化方面，以新技术推广应用为抓手，持续开展更广泛的农科合作，着力打造高效生态农业基地。加大新技术、新品种研发推广力度，积极推进F型杂交小麦研发，加快实现产业化。健全农技推广体系建设，提升农技服务能力。深化与山西农大、中国农科院等科研院所（校）的合作。推进灾害性天气和农作物有害生物预警体系建设，提升农业抵御自然灾害的能力。加强农业机械化技术应用，大力推广节水灌溉、土地深松、精量播种、化肥深施和农作物综合利用等增产增效、节约资源的新技术，不断提高农业的水利化、机械化、信息化水平。大力培育新型职业农民，全年培训1.2万人。

　　经营市场化方面，以农产品市场流通体系建设为抓手，培育农业服务市场化体系，着力打造以品牌为引领的农业发展新格局。加快推进夏县润恒、盐湖晋善晋美、芮城大汉等农业物流园项目建设。抓好农产品产地交易和冷链物流市场、农产品营销网络体系和信息化、农产品营销经纪人队伍、农产品质量安全生产和检测体系等重点。积极推动农产品物联网和电子商务平台等新型业态的培育和发展，加快建立完善畅通的农产品物流体系。大力发展市场化、专业化农业服务企业，在农资、农机、信息、植保、动物防疫等方面完善专业化配套服务，为农业规模化、集约化发展提供有力保障。注重农产品品牌培育，以运城苹果代表中国出口美国为契机，积极对接北上广等高端市场，建设标准化的农产品生产基地，打造一批在全国叫得响的知名农产品品牌和驰名商标。抓好200个“一村一品”精品专业村建设。

　　加快实施“三引六扩、河库成网”大水网建设工程。重点抓好小农水重点县、农村饮水安全、北赵引黄二期、涑水河综合治理、张良调蓄水源库、西塬湖应急加固等水利工程建设。结合小浪底饮水工程，启动中心城市饮用水水质提升工程的前期工作。完成水土流失治理面积27万亩。

　 （三）以工业集群化、集群园区化、园区社区化、社区城镇化、土地规模化、城乡生态化“六化”衔接为路径，“四化”互动推进新型城镇化

　　加强城市规划引领。坚持国民经济和社会发展、城镇规划、国土规划、产业规划和环保规划“五规合一”，突出高端化、生态化、集约化，精心编制运城市发展战略空间规划、中心城市整体设计、绿色建筑集中示范区、中心城区环境卫生、地下空间、城镇社区等各项规划，进一步完善城乡规划体系。积极推进盐临夏一体化进程。

　　推进城市建设。按照“城市功能完善、产业空间拓展、土地集约利用、市民方便宜居”四个目标一个过程实现的要求，在基础设施、公用事业领域积极推行政府和社会资本合作模式。全年计划实施建设项目303项，总投资496.8亿元，今年完成投资162.6亿元。城市功能完善方面，抓好道路、水系、供电、供气、供暖、亮化、通信、垃圾处理、污水处理等基础设施和广场、公园、剧院、学校、医院、体育场馆、公共交通等公共服务设施建设。今年实施项目146项，总投资87亿元。加快与北京首创集团以水务市场化、城市基础设施建设为主要内容的全方位合作。产业空间拓展和土地集约利用方面，注重在城市规划中通过功能完善，进一步创造产业环境。本着增加投资强度、提高产出效益的原则，通过科学的城市设计，运用市场化手段，在旧城改造和建成区提升中，把原来那些容量小、低端化、影响市容的建筑物和产业，通过改造整合，吸引金融、保险、电子商务、总部经济等现代服务业入驻，提升产业档次，拓展产业容量，使土地得到集约高效利用。继续抓好与产业发展相配套的城市主体建筑的招商与建设。市民方便宜居方面，注重功能配套，完善上学、就医、餐饮、购物、休闲、出行等公用服务设施，真正体现方便、温馨、舒适的理念。今年新开工各类保障性住房11353套,基本建成13300套。

　　坚持“四位一体”发展。中心城市启动城建工程152项，总投资348.4亿元，今年计划完成投资106.9亿元。重点抓好河东西街延长线、10个棚户区改造等一批市政基础设施和公共服务项目。大县城实施项目151项，总投资148亿元，今年计划完成投资55.7亿元。小城镇继续抓好15个重点镇建设，引领全市小城镇发展。新农村以“农村人居环境改善”为抓手，重点实施“完善提质、农民安居、环境整治和宜居示范”四项工程，加快推进美丽宜居示范村建设。

　　提升城市管理水平。深化户籍管理制度改革，进一步降低从农村迁入城市的准入条件，建立城乡一体的户口登记制度。强化市民城市意识，持续开展城市环境综合治理，打造宜居宜业宜商的城市环境。

　　加快信息化步伐。坚持以信息化为引领，积极推进“互联网+”行动，加快互联网与传统产业的深度融合，促进传统经济融合创新与转型升级。加快智慧城市建设，全力支持中兴网信在运城投资建设智慧城市项目，抓好云平台建设和智慧政务、智慧教育、智慧食药、智慧城管、智慧医疗五大智慧平台的应用。推进“宽带运城”示范城市建设，完善公共场所无线局域网建设。

　　加强城乡生态建设。加大造林绿化力度，全年完成造林38万亩，新发展干果经济林12万亩。每个县（市、区）至少要建成20个高标准的园林村，高标准完成30公里通道绿化。城市绿化要按照“横有厚度、纵有层次，点成缀、线成景、片成林”的模式，科学规划实施，全面提高绿化水平和品位，新增绿化面积100万平方米。深入开展栽植“家庭亲情林”活动，让种树成为运城人的一种时尚、一种习惯、一种情怀。认真落实新《环保法》。大力开展资源综合利用和清洁生产。抓好循环经济试点市建设。加强水环境治理，强化饮用水水源地环境监管，城市集中式饮用水水源地水质达标率达到100%。抓好重点流域、“一河一渠”环境综合整治。深入开展大气污染防治。加大淘汰落后产能力度，全面完成“十二五”减排任务。

　 （四）以科技创新、金融振兴和民营经济发展为突破，推进重点领域改革

　　加快推进“三大突破”。运城经济的主体是民营经济，科技创新和金融振兴重在服务和助推民营经济发展。推动科技创新，要落实国家创新驱动发展战略运城行动计划，组织引导集群内企业与产业对口的科研单位、行业协会对接，建立技术战略联盟，促进科技成果转化，提升科技对经济增长的贡献率。加强知识产权保护。加快金融振兴，要落实金融支持“三个方案”实施意见，强化全社会的现代金融意识，特别是领导干部和企业家要把学金融、懂金融、用金融作为经济发展新常态下的一门必修课。政府、企业、金融业携手推进多层次资本市场建设。积极推进中小企业股份制改造，鼓励支持企业直接上市融资，对已经选出的100家企业要分期分批完成股份制改造。创优金融生态环境，防范和化解金融风险。壮大民营经济，要按照“三个方案”的要求，对九大产业集群、农业种养加企业以及现代服务业等各类民营企业，加大扶持力度。加快中小微企业创业基地、中小微企业服务联盟和运城市中小微企业综合服务窗口平台建设，大力扶持市场前景好、科技含量高、发展潜力大的中小微企业。

　　深入推进政府职能转变。加快行政审批制度改革。承接落实好省政府取消、下放和调整的行政审批事项。落实好应向县级下放审批事项，开展非行政许可审批事项的专项清理工作，编制完成政府部门权力清单。积极推进政府购买公共服务，凡是通过市场行为能够解决的问题一律交由市场解决，严格控制新增政府机构和编制，倒逼政府职能转变，提升服务水平。推进城市建设投融资体制机制改革创新，推广“PPP模式”，防范和化解政府债务风险。深化工商注册登记制度改革，切实为市场经营主体松绑。加快医药卫生体制改革和市县两级公立医院改革。深入推进事业单位分类改革和综合行政执法体制改革。进一步深化社会组织管理制度改革，加快行业协会、商会与行政机关脱钩。深化文化体制改革。完成公务用车制度改革。加快不动产统一登记制度改革，年底前完成市县两级不动产登记职责整合及机构组建。

　　创新财政资金使用分配机制。围绕“三个方案”编制财政预算，今年市本级财政共整合安排“三个方案”资金6.2亿元，其中，工业集群化2亿元，农业现代化1.6亿元，新型城镇化2.6亿元。要通过“争取、撬动、整合、盘活”等途径，落实好工业集群化、农业现代化、新型城镇化、文化旅游产业以及“万众创业”等产业引导基金，进一步健全完善企业应急转贷专项资金、信贷风险补偿基金等，引导更多社会资本投入“三个方案”项目建设，放大财政资金的效应，扶持企业发展壮大。

　 （五）以招商引资和项目建设为抓手，全力扩大对外开放

　　坚持不懈抓好招商引资。招商引资是推动经济社会发展的常态性工作，是推进“三个方案”落实的主要动力，是衡量一级政府工作能力和成效的重要标尺。要按照“三个方案”的总体要求和年度目标，持续开展集群化、专业化、精准化、产业链和小分队招商。全年招商引资到位资金力争达到600亿元以上。

　　毫不放松推进项目建设。坚持储备、签约、落地、开工、建设、投产“六位一体”工作机制，严格落实项目包联责任制。大运重卡、盐湖区飞宇铝型材、同誉铝轮毂二期、稷山阳煤丰喜焦炉煤气综合利用、同煤寺塔120万吨矿井，永济2×35万千瓦电厂等一批在建项目，要确保如期竣工投产；蒙西至华中铁路运城段、华泽铝电低热值煤发电等一批基础设施项目，年内要开工建设。全年实施重点项目243项，完成投资1011亿元。

　　持之以恒深化区域合作。抢抓“一带一路”建设新机遇，加快推进晋陕豫黄河金三角规划实施,深化三省四市合作，巩固和扩大在果业、旅游、医疗、质监等方面的合作成果。积极争取黄河综合治理、铝工业基地建设、“古中国”文化旅游、现代特色农业示范区、金三角区域现代金融中心等一批重大事项纳入国家“十三五”规划。推进转型综改试验区建设，抓好省级转型综改重大项目建设，稳步推进扩权强镇试点。深化与杭州、西安、黑河等友好城市和长三角、珠三角、环渤海等区域的合作交流。大力发展飞地经济和总部经济。完善海关、商检、边检等基础设施，推进运城航空口岸开放工作。

　（六）以打造“古中国”为标识的国际旅游目的地为目标，加快文化旅游产业和现代服务业发展

　　加快旅游产业发展。充分发挥我市文化旅游资源优势，加大创意策划和市场开发力度，加强与北京、西安、洛阳等城市的旅游合作。强化文化旅游产品营销，打造具有运城特色的旅游情景剧，培养一批高素质的导游队伍，提升景区景点管理和服务水平。加强“古中国”国际旅游目的地宣传推介，提高运城“古中国”品牌国际影响力。开通运城高铁站至景区直通车。继续推行运城人游运城“一卡通”，实现“一卡通”智能化和便利化。加快智慧旅游建设步伐，争取10月底前实现4A级景区智慧旅游全覆盖。积极推进关圣文化建筑群申遗工作，做好建筑群文物本体保护修缮工作，加大周边环境整治力度。全年接待游客突破4000万人次，旅游收入突破300亿元，同比增长16%。

　　大力发展文化产业。深入挖掘“古中国”的文化内涵，大力宣传运城作为“古中国”源头所蕴含的文化基因价值，加大文化产业与旅游产业融合发展的深度。扩大关公文化、盐文化、根祖文化等对旅游产业的带动作用。继续抓好盐湖清尚、夏县宇达、稷山大佛寺、芮城永乐宫、万荣李家大院等文化产业园建设。加快推进以“三个禁门”为中心的河东盐池文物复修和保护工程。鼓励和支持社会力量投资文化产业。制定和实施文化小微企业孵化计划，培育和扶持文化骨干企业。

　　着力壮大现代服务业。大力发展电子商务，培育电子商务人才，推动电子商务企业集聚。支持星河创客、寰烁科技等电子商务平台健康发展。大力发展现代物流业，推进黄河金三角智慧物流城建设。积极培育新兴业态，推动金融服务、信息服务、检验检测等生产性服务业和文化体育、健康养老、家政服务、社区服务等生活性服务业发展。

　 （七）以保障和改善民生为根本，全面发展社会事业

　　大力发展教育事业。尊师是社会的良知，重教是政府的德行。我们要不断加大财政投入力度，确保我市教育始终走在全省前列。加大普惠性幼儿园建设力度。新改扩建28所公办标准化幼儿园，新建改建42所农村幼儿园。推进义务教育办学模式改革，全面改善义务教育薄弱学校和寄宿制学校基本办学条件，完成盐湖区、夏县农村薄弱学校改造任务。加大城乡教师交流、校长轮岗力度，区域内义务教育阶段学校校长、教师交流实现全覆盖。紧紧围绕“三个方案”，大力发展现代职业教育，加快组建汽车、文化旅游、家居产业、卫生保健、制版印刷等职业教育集团，为经济转型发展培养应用型、技能型人才。落实特殊教育提升计划，办好特殊教育学校。积极适应经济社会发展需要，提升高等教育质量和水平。

　　加快发展医疗卫生事业。全面落实基层医疗卫生机构综合改革各项政策，提升新农合保障水平。人均基本公共卫生服务经费财政补助标准由35元提高到40元。为农村60岁以上老年人实行免费体检。加强农村医生队伍建设，提高基层医疗卫生机构服务能力。加快推进市第一医院、市儿童医院和运城健康城项目建设。

　　加强社会保障工作。积极推进机关事业养老保险制度改革。企业退休人员基本养老金提高10%以上。城乡居民基本养老保险基础养老金最低标准每人每月增加15元，达到80元，城镇居民医保人均财政补助标准由320元提高到380元。进一步提高城乡低保、农村五保对象补助水平。兑现落实省政府调资政策。全面治理拖欠农民工工资问题，健全农民工工资支付保障机制，决不允许再有拖欠农民工工资的现象在运城的土地上发生。新建老年人日间照料中心185个。发展社会福利慈善事业。全面落实各项救助制度，确保困难群众求助有门、受助及时。

　　落实就业创业政策。统筹抓好高校毕业生、农村转移劳动力、城镇困难人员、企业下岗职工、退役军人等群体的就业工作，确保全年城镇新增就业5.6万人，转移农村劳动力7.14万人。大力推进“大众创业、万众创新”，把运城人深厚的文化底蕴转化为创新创业的智慧。在全社会倡导创业文化，鼓励运城籍成功人士回乡创业，让“学而优则商”成为运城人，特别是年轻人新的价值取向和人生追求。加快国家级高技能人才培训基地建设，培训各类职业技能人才9.8万人，新增高技能人才5300人。

　　深入开展文化体育惠民活动。加强公共文化服务体系建设，组织举办丰富多彩的文化活动。繁荣文艺创作，精心打造体现“古中国”文化底蕴的优秀艺术作品。继续推进“戏曲惠民”活动。加快河东会堂市场化改造工程，河东大剧院年内全面开工建设。充分发挥各级各类图书馆、文化中心、农家书屋、社区书社作用，倡导全民阅读，打造“书香运城”，让阅读成为运城人的一种生活方式和行为习惯。搞好非物质文化遗产的挖掘、保护和利用。完成全市22947户广播电视直播卫星户户通任务。强化文化市场执法管理和“扫黄打非”工作。大力发展体育产业，广泛开展全民健身活动，办好我市第四届运动会。加强和改进档案工作，推进档案馆（库、室）标准化建设。

　　抓好安全生产工作。建立健全“党政同责、一岗双责、齐抓共管”的安全生产责任体系，落实政府安全监管责任，强化企业安全生产主体责任。坚持依法治安，加强重点领域安全监管，深入开展重点行业和领域的安全生产专项整治。强化食品药品安全监管，继续推进食品安全放心城市创建工作，严厉打击涉食涉药违法行为，坚决取缔食品加工黑作坊、黑窝点，确保人民群众饮食用药安全。强化责任追究，严格落实安全生产目标责任考核“一票否决制”，坚决遏制重特大事故、减少一般性事故，努力促进安全生产形势由稳定好转向根本好转迈进。

　　要认真调查研究，全力做好“十三五”规划编制工作，科学谋划好运城未来五年的发展蓝图。

　 （八）以坚持公平正义为核心，努力维护社会和谐稳定

　　加强民主法治建设。自觉接受人大及其常委会监督，认真执行人大各项决议。积极支持人民政协履行政治协商、民主监督和参政议政职能。坚持重大事项向人大报告、向政协通报和重大决策提交人大审议制度。充分听取各民主党派、工商联、人民团体和社会各界意见，认真办理人大代表建议和政协委员提案。积极支持人民法院、检察院依法履行职责。建设完备的公共法律服务体系，普遍建立县乡公共法律服务中心，扩大法律援助覆盖面。深入推进“六五”普法，强化全民法治观念，加快依法治市进程。

　　加强精神文明建设。充分发挥河东传统文化优势，培育和践行社会主义核心价值观。加强选树道德模范工作，倡导新风尚，凝聚正能量。大力加强社会公德、职业道德、家庭美德、个人品德建设，提升运城人“睿智明理、勤勉敬事、仗义好胜”的人文形象。我们的干部心中要充满正义力量、道德力量、人格力量，充分发挥榜样作用，以优良的党风政风，影响民风，引领社风。坚持正确的舆论导向，加强网络舆情管理。加强未成年人思想道德建设，广泛开展群众性精神文明创建活动，不断提升市民文明素质和社会文明程度。

　　加强和创新社会治理。善于综合运用经济、法律、行政、道德、舆论以及社会心理等多种办法，理顺人心，做到事态平、人心平、社会太平。推动政府治理和社会自我调节、居民自治良性互动，建立完善社会调处化解矛盾纠纷综合机制。加强信访工作，创新体制机制，健全完善畅通有序的诉求表达渠道，积极解决人民群众的合理诉求。推进“平安运城”建设，创新立体化防控体系。加强社会治安综合治理，深入开展“打黑除恶”斗争和社区矫正工作，有效防范和及时处置突发性群体事件。发展专业社会工作和志愿服务工作。加强应急管理和防灾减灾能力建设。增强全民国防观念，加强国防后备力量建设，做好优抚安置和新一轮全国双拥模范城创建工作，推动军民融合深度发展。支持工、青、妇工作，充分发挥群团组织的作用，发展儿童、老龄、残疾人和红十字会等事业，做好民族宗教、外事侨务、文物保护、港澳台等工作，加强气象、地震、科普、方志等工作。

　　三、全面加强政府自身建设

　　各位代表，政府自身建设，既是政府体制机制的建设，也是政府公务人员的自身建设。我们要按照“三严三实”的要求，坚持为民务实清廉，全面加强和改进政府自身建设。

　 （一）强化法治意识，保障行政效率

　　政府的权力是公共权力，是由人民赋予我们并代表人民行使对公共事务进行管理的权力。公务人员行使公共权力必须在法律规定的范围之内，超越了法律的界限，就是对权力的滥用，就要受到法律的制裁。依法行政，就要做到法定职责必须为，法无授权不可为，就是要按照“六权治本”的精神，制定权力清单、责任清单和负面清单，编制行政职权运行流程图和行政职权运行风险防控图，构建公共资源交易平台和政务服务平台，严格执法程序，规范执法行为。用依法行政保障公共权力运行效率，维护世道人心，从而带动全社会形成依法谋事、依法办事、依法论事、依法论人、依法自律的法治文化氛围。

　 （二）强化学习意识，提高创新能力

　　思维方式决定发展视野和发展格局。要按照市委加强“三个建设”、争做“三个楷模”、提升“三个能力”的要求，加强学习型政府建设。坚持一月一次“政府大讲堂”活动，在各级领导干部和公务员队伍中，倡导并形成浓厚的学习风气，进而引领全体干部创新思维方式、领导方式和工作方式，以开放的视野和创新的精神谋划和推进运城发展。

　 （三）强化自律意识，建设廉洁政府

　　要认真贯彻中央、省反腐倡廉各项决策部署，全面落实政府系统党风廉政建设责任制和党委（党组）主体责任，切实把主体责任放在心上，抓在手上，扛在肩上。围绕土地、矿产、交通、房地产等重点领域，围绕教育、医疗、环保、社保、涉农等社会民生领域，围绕资源配置、工程招标、政府采购、资金分配等关键环节，围绕行政审批违规问题和滥用审批权问题，扎实开展专项整治。特别是在土地、财政资金、人事和项目实施等方面，坚守底线，从严把关，严禁任何人干涉工程招投标，对涉及财政资金使用的领域和工程实行全覆盖、全过程的审计监督。严肃查处违法违纪案件，坚决纠正部门和行业不正之风。在政府部门坚决遏制庸俗的人情往来，努力营造简单清爽、纯洁健康、团结和谐的同志关系和工作氛围。

　 （四）强化落实意识，改进工作作风

　　落实能力是对各级领导干部行政能力和工作水平的现实考验。要积极构建部门之间推进工作的横向信息反馈、纵向动态平衡，科学有效、协调顺畅的工作机制。坚持一级抓一级、层层抓落实，真正形成工作部署自上而下、工作落实从下到上，层层反馈、层层负责的工作格局。加强督查落实和目标责任考核，完善考核体系，努力将各项目标任务明确到单位、落实到人头、具体到项目。特别是要把落实“三个方案”作为干部干事创业的重要平台，作为识别、锻炼和检验干部的“赛马场”，在推动落实“三个方案”的实践中发现人才、储备人才、培养人才、使用人才。以只争朝夕的状态、实事求是的态度、功成不必在我的境界和抓铁有痕、踏石留印的作风，推动各项工作不断实现新的突破。

　　各位代表，2015年已经时间过半，完成全年各项目标任务繁重而艰巨。让我们紧密团结在以习近平同志为总书记的党中央周围，在省委、省政府和市委的正确领导下，振奋精神、坚定信心，团结拼搏、开拓奋进，着力落实“三个方案”，全力推动“六大发展”，为全面完成“十二五”规划目标，开创弊革风清、富民强市新局面而努力奋斗！
